# Opening the black box of spring water microbiology from alpine karst aquifers to support proactive drinking water resource management

**DOI:** 10.1002/wat2.1282

**Published:** 2018-03-09

**Authors:** Domenico Savio, Philipp Stadler, Georg H. Reischer, Alexander K.T. Kirschner, Katalin Demeter, Rita Linke, Alfred P. Blaschke, Regina Sommer, Ulrich Szewzyk, Inés C. Wilhartitz, Robert L. Mach, Hermann Stadler, Andreas H. Farnleitner

**Affiliations:** ^1^ Division Water Quality and Health Department Pharmacology, Physiology and Microbiology, Karl Landsteiner University of Health Sciences Krems a. d. Donau Austria; ^2^ Centre for Water Resource Systems Technische Universität Wien Vienna Austria; ^3^ Institute for Water Quality, Resource and Waste Management Technische Universität Wien Vienna Austria; ^4^ Institute of Chemical, Environmental & Bioscience Engineering, Research Group Environmental Microbiology and Molecular Diagnostics 166/5/3, Technische Universität Wien Vienna Austria; ^5^ Interuniversity Cooperation Centre for Water and Health, www.waterandhealth.at; ^6^ Unit Water Hygiene, Institute for Hygiene and Applied Immunology Medical University of Vienna Vienna Austria; ^7^ Institute of Hydraulic Engineering and Water Resources Management Technische Universität Wien Vienna Austria; ^8^ Department of Environmental Technology Technical University of Berlin Berlin Germany; ^9^ Department of Environmental Microbiology Eawag, Swiss Federal Institute of Aquatic Science and Technology Dübendorf Switzerland; ^10^ Department for Water Resources Management and Environmental Analytics Institute for Water, Energy and Sustainability, Joanneum Research, Graz Austria

**Keywords:** alpine karst aquifers, spring water microbiology, natural spring water microbes, external fecal pollution, drinking water resource protection, water abstraction management, online water quality monitoring, fecal indicator, microbial fecal source tracking, microbial risk assessment, future challenges

## Abstract

Over the past 15 years, pioneering interdisciplinary research has been performed on the microbiology of hydrogeologically well‐defined alpine karst springs located in the Northern Calcareous Alps (NCA) of Austria. This article gives an overview on these activities and links them to other relevant research. Results from the NCA springs and comparable sites revealed that spring water harbors abundant natural microbial communities even in aquifers with high water residence times and the absence of immediate surface influence. Apparently, hydrogeology has a strong impact on the concentration and size of the observed microbes, and total cell counts (TCC) were suggested as a useful means for spring type classification. Measurement of microbial activities at the NCA springs revealed extremely low microbial growth rates in the base flow component of the studied spring waters and indicated the importance of biofilm‐associated microbial activities in sediments and on rock surfaces. Based on genetic analysis, the autochthonous microbial endokarst community (AMEC) versus transient microbial endokarst community (TMEC) concept was proposed for the NCA springs, and further details within this overview article are given to prompt its future evaluation. In this regard, it is well known that during high‐discharge situations, surface‐associated microbes and nutrients such as from soil habitats or human settlements—potentially containing fecal‐associated pathogens as the most critical water‐quality hazard—may be rapidly flushed into vulnerable karst aquifers. In this context, a framework for the comprehensive analysis of microbial pollution has been proposed for the NCA springs to support the sustainable management of drinking water safety in accordance with recent World Health Organization guidelines. Near‐real‐time online water quality monitoring, microbial source tracking (MST) and MST‐guided quantitative microbial‐risk assessment (QMRA) are examples of the proposed analytical tools. In this context, this overview article also provides a short introduction to recently emerging methodologies in microbiological diagnostics to support reading for the practitioner. Finally, the article highlights future research and development needs.

This article is categorized under:

1 Engineering Water > Water, Health, and Sanitation
2 Science of Water > Water Extremes
3 Water and Life > Nature of Freshwater Ecosystems

**Abbreviations**
AMEC: Autochthonous microbial endokarst community
DKAS: Dolomite karst aquifer spring
DNA: Deoxyribonucleic acid
EM: Epifluorescence microscopy
FCM: Flow cytometry
FISH: Fluorescence in situ hybridization
HPC: Heterotrophic plate counts
HTS: High‐throughput sequencing
LKAS: Limestone karst aquifer spring
MST: Microbial source tracking
NCA: Northern Calcareous Alps
PSP: Pollution source profiles
QMRA: Quantitative microbial‐risk assessment
qPCR: Quantitative real‐time polymerase chain reaction
RNA: Ribonucleic acids
SFIB: Standard fecal indicator bacteria
TCC: Total cell counts/concentrations
TMEC: Transient microbial endokarst community
VBNC: viable but not cultivable

## 1 INTRODUCTION

Alpine karst aquifers play a vital role in the drinking water supply in many regions throughout the world (Ford & Williams, 2007a). For example, at least 95% of the drinking water demand of the City of Vienna is provided by such ground water resources, and more than 1.6 million Viennese inhabitants appreciate the high‐quality drinking water from the nearby mountain regions that is directly delivered to their households (Griebler & Avramov, 2015; ten Brink et al., 2011). Maintaining the highest quality standards requires coordinated and information‐driven efforts, including the sustainable protection of the catchments, optimized spring‐abstraction management, and sufficient final treatment. For example, it is well known that during precipitation events, karst springs can be very rapidly influenced by microbes originating from fecal pollution from the surface (Bucci, Petrella, Naclerio, Allocca, & Celico, 2015; Pronk, Goldscheider, & Zopfi, 2006; Stadler et al., 2008). Thus, an adequate understanding of the potential factors affecting microbiological water quality is of utmost importance to guide a target‐oriented and sustainable water resource management. Until recently, however, in particular the information on the microbiological water quality of alpine karst springs has mainly been sourced from a fragmentary puzzle of routinely performed surveillance activities. In‐depth knowledge on the different aspects of microbiological water quality was largely missing for alpine karst springs. Thus, intensive research activities on the microbiology of alpine karst springs were prompted at the Northern Calcareous Alps (NCA) of Austria at the start of the new millennium. Thereby, a detailed knowledge on the hydrogeological background of the studied alpine karst model systems was regarded as a basic and essential requirement for the interdisciplinary research efforts.

**Figure 1:**
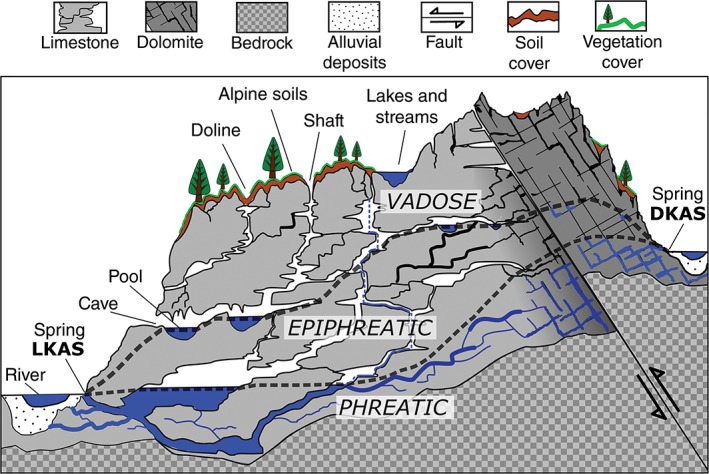
Schematic illustration of alpine karst systems with differing hydrogeological backgrounds, with “LKAS” representing a typical spring of a limestone karst aquifer, and “DKAS” representing a typical spring of a dolomite karst aquifer system. Dashed lines indicate the transition zones between the saturated phreatic, the temporarily flooded epiphreatic, and the unsaturated vadose zones

The karst springs in focus of this overview article are typical springs of the alpine karst regions (cf. Figure 1). The term *alpine karst*, as used in this article, refers to karst formations formed in areas of high altitude and relief (Field, 1999). The definition of *alpine karst* is therefore not geographically restricted to the karst landscapes of the Alps (Ford, 1971; Günay, 2010; Ozyurt & Bayari, 2008), it generally relates to karst systems of regions determined by mountainous topography and high altitude climate (Figure 1). In large parts, alpine karst systems are *vulnerable* to fecal contamination, where vegetation and soil covers are thin or not abundant. Point recharge in respective areas may be enhanced due to overlaying geological layers, as well as thrust and fold structures (Goldscheider, 2005, 2010; White, 1969). Distinct hydrograph peaks during early summer months caused by snow melt in the hydrogeological catchment area are symptomatic for karst springs draining alpine karst systems of temperate latitudes (Stadler et al., 2008, 2010).

The aim of this article is to give an overview on the developed methodologies and the established knowledge on the NCA spring water microbiology that has been studied over more than 15 years. The results will also be set into context of literature from other relevant areas and future research requirements. Special emphasis is also given to components with importance for the water quality management of drinking water supplies. It has to be mentioned that this work is not designed as a conventional in‐depth review on general karst microbiology. To keep this work digestible for a practitioner in the field of water resource management, also a short introduction to recently emerging methodologies in the field of microbiological diagnostics will be given.

## 2 ESTABLISHED STANDARD AND EMERGING METHODS IN MICROBIOLOGICAL DIAGNOSTICS

**Figure 2:**
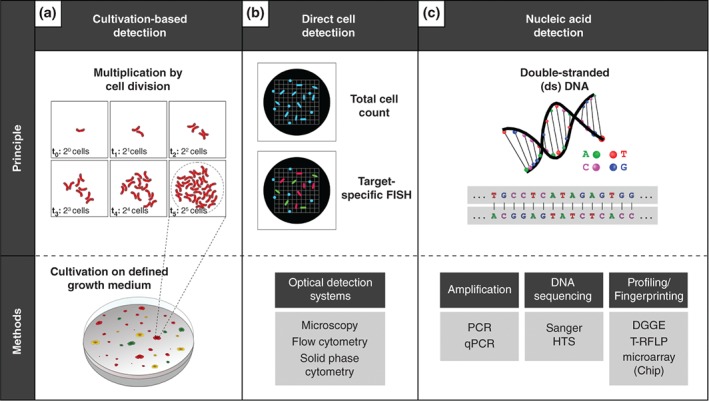
Figure summarizing methods commonly applied for the specific or nonspecific detection or quantification of microorganisms in environmental samples (for details see main text). Panel (a) schematically illustrates the traditional cultivation‐based method commonly applied in water quality monitoring, which works by growing and detecting cultivable microbes on artificial growth media. Panel (b) shows the principle as well as the most commonly used applications for the direct optical detection of microbial cells. The upper scheme illustrates the principle for detecting total cell counts based on nonspecific fluorescence dyes. The graphic below illustrates the specific labeling of particular groups of microbes. Panel (c) summarizes commonly applied methods for the (quantitative) detection of microorganisms by DNA/RNA amplification, DNA/RNA sequencing, and community profiling. The figure shows a schematic illustration of a double‐stranded DNA helix (top) and the corresponding DNA‐sequence (below). DNA, deoxyribonucleic acid; a, adenine; T, thymine; C, cytosine; G, guanine; (q)PCR, (quantitative real‐time) polymerase‐chain‐reaction; HTS, high‐throughput sequencing; DGGE, denaturing gradient gel electrophoresis; T‐RFLP, terminal restriction fragment length polymorphism; and FISH, fluorescence in situ hybridization

At a basic level, microorganisms are defined as organisms that are invisible to the naked eye (<150 μm). The first methods—based on cultivation on artificial growth media—were introduced in the second half of the 19th century (Koch, 1882). Thereby, the detection is based on cell division, which results in the formation of colonies that are large enough to be detected by the naked eye (Figure 2a). Even today, most standardized detection methods for health‐related water quality testing such as for the detection of fecal indicator bacteria are based on this highly sensitive and straightforward enumeration principle (International Organisation for Standardisation, 1999, 2000a, 2000b; Reasoner & Geldreich, 1985). However, most microbes occurring in aquatic habitats cannot be grown on standard cultivation media as they require very specialized and often unknown growth conditions (Staley & Konopka, 1985). Besides missing knowledge on the optimal growth conditions of most bacteria, additional challenges in the cultivation may arise from bacteria being in a dormant or so‐called viable but not cultivable (VBNC)‐state (Königs, Flemming, & Wingender, 2015; Li, Mendis, Trigui, Oliver, & Faucher, 2014; Oliver, 2010; Xu et al., 1982). In the field of microbiological water quality monitoring, the difficulties to cultivate or detect particular microbes of interest (e.g., intestinal pathogens) resulted in the development of the so‐called indicator bacteria concept (Bonde, 1966), where alternative organisms are grown and used as a proxy for the microorganism of interest.

In contrast to cultivation‐based methods, direct cell‐based investigation methods such as the traditional microscopy‐based approach overcome the necessity of growth, using optical systems to detect or quantify cells directly in the water phase or on a filter surface after filtration or enrichment (Figure 2b). The latest developments of automated optical systems such as flow cytometry (FCM) or solid‐phase cytometry even support the automated enumeration of the total number of microbial cells (total cell counts, TCC) in water samples (Besmer et al., 2014, 2016; Hammes et al., 2008; Hoefel, Grooby, Monis, Andrews, & Saint, 2003; Page et al., 2017; Riepl et al., 2011). In a very recent review, Van Nevel et al. (2017) even initiated a discussion on whether FCM could replace a standard cultivation‐based method (heterotrophic plate counts, HPC) in routine water quality monitoring. One major challenge when working with environmental microorganisms is that their variability in terms of morphological appearance and shape is highly restricted (Young, 2007). Consequently, with exceptions, different groups or species cannot be reliably differentiated by conventional cell‐based detection methods such as FCM or traditional epifluorescence microscopy (EM). To distinguish or identify particular microbes of interest, microscopy, or FCM has to act in concert with specific cell‐labeling methods (DeLong, Wickham, & Pace, 1989; Pickup, 1991; Schloter, Aßmus, & Hartmann, 1995). Many labeling techniques such as fluorescence in situ hybridization (FISH; Amann, Krumholz, & Stahl, 1990; Pernthaler, Pernthaler, & Amann, 2002; Wagner & Haider, 2012) are based on the power of targeting specific intracellular nucleic acid molecules (Figure 2b, see the following paragraph).

As the most essential biomolecule in all organisms, DNA (deoxyribonucleic acid) contains the genetic information (“construction plan”) of every single cell. Its informational content is determined by the sequential order of the building blocks (nucleotides) Guanine (G), Adenine (A), Cytosine (C), and Thymine (T), forming a four‐letter‐based “code” commonly referred to as a “DNA sequence” (Figure 2c). A typical bacterial genome, for example, is comprised of 10^6^–10^7^ nucleotides, which equals approximately 1–10 MB when translated into computer language. This quantity highlights the enormous informational capacity stored in a tiny molecule of approximately 2 nm in diameter and approximately 1.4 mm in length. This encoded information is used for the specific identification and analysis of microbes by nucleic acid‐targeting technologies. Potential target nucleic acids include not only DNA but also RNA—a second type of nucleic acid. In contrast to the above‐mentioned labeling techniques, short fragments of DNA/RNA molecules (up to a maximum of several hundred base pairs in length) can also be specifically detected by nucleic acid‐based amplification techniques. For example, one of the commonly applied approaches to quantify microbes based on their characteristic DNA fragments is the so‐called quantitative real‐time polymerase chain reaction (qPCR; Figure 2c). This method is also commonly applied in so‐called microbial source tracking (MST), where pollution source‐associated bacteria (such as bacteria occurring in particular animals like ruminants or pigs) are targeted to investigate their host's contribution to fecal pollution. For a comprehensive review of currently available MST tools targeting bacteria of the order *Bacteroidales*, see the work by Ahmed, Hughes, and Harwood (2016). In contrast to the quantification of known DNA targets, the revolutionary invention of nucleic acid sequencing allows to “read” the informative content stored within the four‐letter code of previously unknown DNA/RNA (Sanger, Nicklen, & Coulson, 1977). This capability to read the informative content of DNA has soon also been utilized in combination with so called “community profiling” or “fingerprinting” methods, which until recently were commonly applied to characterize microbial populations in environmental samples (Figure 2c). These methods generate characteristic patterns from bacterial communities based on the differences in nucleotide composition and/or the length of the investigated nucleic acid fragments, and further enable their isolation and individual analysis by DNA sequencing (Burtscher, Zibuschka, Mach, Lindner, & Farnleitner, 2009; Farnleitner et al., 2000, 2004; Lee, Zo, & Kim, 1996; Liu, Marsh, Cheng, & Forney, 1997; Muyzer, de Waal, & Uitterlinden, 1993; Muyzer & Smalla, 1998). Another very recent and ground‐breaking invention was the rise of novel high‐throughput sequencing (HTS) techniques, which once again revolutionized the entire field of bioscience by providing the capability to analyze millions of nucleic acid sequences in parallel within a few hours (Rothberg & Leamon, 2008). As a consequence, nucleic acid sequence data analysis based on bioinformatical tools emerged as a new subdiscipline in microbiology, and the required know‐how and processing time to handle the huge amounts of generated data are increasingly considered a “bottleneck” of large field investigations (Carlos, Tang, & Pei, 2012; Scholz, Lo, & Chain, 2012). The application of this novel methodology to water samples of all kinds enables an in‐depth characterization of their microbial communities’ composition, and has recently also been applied in combination with FCM to study the temporal community dynamics in the context of the biostability^2^ of water (Prest et al., 2014).

Besides these cell‐based and molecular biological methods, other available methods increasingly applied in water quality monitoring such as adenosine triphosphate (ATP) detection (Vang, Corfitzen, Smith, & Albrechtsen, 2014) make a trade‐off between reduced specificity but faster detection/higher time resolution (Højris, Christensen, Albrechtsen, Smith, & Dahlqvist, 2016; Lopez‐Roldan, Tusell, Cortina, Courtois, & Cortina, 2013).

## 3 THE GENERAL CHARACTERISTICS OF KARST SYSTEMS

*Karst* denominates a specific kind of landscape with soluble rocks in the underground, such as carbonates (e.g., limestone, dolomite), that developed an extensive underground water system containing complex cavity‐ and cave structures (Ford & Williams, 2007b; cf. Figure 1). Karst comprises terrains with distinct hydrology and landforms, characterized by sinking streams, caves, enclosed depressions (e.g., dolines), fluted rock outcrops and large springs (Ford & Williams, 2007b; Kresic & Stevanovic, 2010). Karst generation is determined not only by the rock solubility, but rock structures, such as thrusts and folds and stratigraphy, as well as leveling of the receiving water bodies are also significant (Ford, 1971; Ford & Williams, 2007b; White, 1969). Karst ground water systems evolve over time, distinguishing it from other groundwater systems, as karst water flow becomes increasingly turbulent due to the progressive solutional enlargement of void space (Ford & Williams, 2007b). Consequently, equations that can be used to describe laminar water flow in porous aquifers become inapplicable to karst (Ford & Williams, 2007b). As karst landforms develop, the karst water level is striving for the level of the receiving water body. It is therefore that a vertical sequence of caves and springs can often be found on hill slopes of karst landscapes. Lower situated springs draining the phreatic zone are constantly discharging (Figure 1). During hydrological events, higher situated springs act as the karst system's overflow and are draining the epiphreatic zone (cf. Figure 1). Such springs fall dry during base flow conditions. The complex and hydraulically connected network of underground voids, cavities and caves determines the hydrogeological catchment. The hydrogeological catchment of a karst spring is not determined by surface topography and can therefore exceed the orographical catchment area significantly (Goldscheider, 2005; Stadler et al., 2008). Karst systems often show a prompt and direct hydrological response to precipitation events, resulting in high‐discharge dynamics of springs (Kresic & Bonacci, 2010; Stadler et al., 2010). Thin or absent soil covers in combination with extended rock cavities prevent an effective natural purification of infiltrating water. Surface‐associated pollution can be introduced almost unhindered into the groundwater (Eckhardt, 2010; Goldscheider, 2010; Kresic, 2010). Therefore, such karst landscapes are highly vulnerable systems in which surface‐associated contamination has rapid and direct impact on the water quality (Farnleitner et al., 2005; Sinreich, Pronk, & Kozel, 2014).

## 4 SELECTED MODEL CATCHMENTS, HYDROGEOLOGY, AND HYDROLOGY‐GUIDED INVESTIGATIONS

The five selected alpine karst springs in focus of this overview article are situated at different locations in the eastern part of the NCA of Austria, with catchment sizes ranging from 4 to 60 km^2^ (Farnleitner et al., 2005, 2011; Reischer et al., 2011). Catchments reach a maximum altitude of approximately 2,300 m above sea level (m.a.s.l.), with wide plateaus and steep slopes. Springs are situated close to their recipients at altitudes between 500 and 800 m.a.s.l. Alpine pastures, krummholz areas and alpine forests are the main land‐use features (Dirnböck, Dullinger, Gottfried, & Grabherr, 1999). Tourism activities (hiking and mountaineering), agriculture based on summer pastures (mainly cattle), and wildlife represent the potential (fecal) pollution sources (Reischer et al., 2011). The lithology of the Triassic sediments ranges from different limestones (Wettersteinkalk and Dachsteinkalk) to dolomite (mainly Wettersteindolomit; Bryda, n.d.). Although the investigated areas feature intensive karstification, direct access to the karst aquifer and its water table is not provided. Caves are widely scattered, but spacious caves in direct connection with the investigated springs are not present (Plan, Hartmann, & Hartmann, 2016).

The four limestone karst aquifer springs (LKAS2, 4, 6, 8) and one dolomite karst aquifer spring (DKAS1) in the NCA region were investigated in detail for several years (for a schematic cross section, see Figure 1). Infield online sensors installed at all selected spring sites for the continuous measurement of discharge and other physicochemical parameters (temperature, pH, electrical conductivity, turbidity, spectroscopic absorption at 254 nm, etc.) enabled a detailed hydrological characterization of the springs (Stadler et al., 2008, 2009, 2010). The mean discharges of the studied springs varied according to the hydrogeology, catchment size, and altitude, and ranged from 250 to 5,100 L per second during the investigation period. The estimated mean water residence time of the LKAS systems ranged from 0.5 to 1.5 years, whereas the residence time for DKAS1 was on the order of 22 years (Stadler & Strobl, 1997). Hydrology‐guided microbiological investigations were supported by automated sampling using data communication via low‐earth‐orbit (LEO) satellites (Stadler et al., 2008). A nested sampling design was developed to representatively cover the base flow conditions as well as periods of increased precipitation during the annual investigations. In addition, detailed precipitation event‐triggered sampling efforts during shorter time periods complemented the nested sampling design (Farnleitner et al., 2011).

## 5 OCCURRENCE OF MICROBIAL COMMUNITIES IN SPRING WATER OF ALPINE KARST AQUIFERS

Microbes are essential for energy and matter flux in the environment (e.g., degradation of organic matter). Their ubiquitous occurrence is only limited by a few environmental factors such as extremely alkaline/acidic conditions (pH >12.5 and <0.5) or temperature (>121°C; Hendry, 2006). Thus, microbes are expected to inhabit any accessible habitat on earth within these limits of life. However, in contrast to many other habitats (Whitman, Coleman, & Wiebe, 1998), until recently, hardly any information was available on the occurrence of microbes in spring water from (alpine) karst aquifers (Griebler & Lueders, 2009). The only exceptions were a few cultivation‐based studies (Menne, 1999; Pavuza, 1994).

### 5.1 Abundance and variability of total microbial cell counts

**Figure 3:**
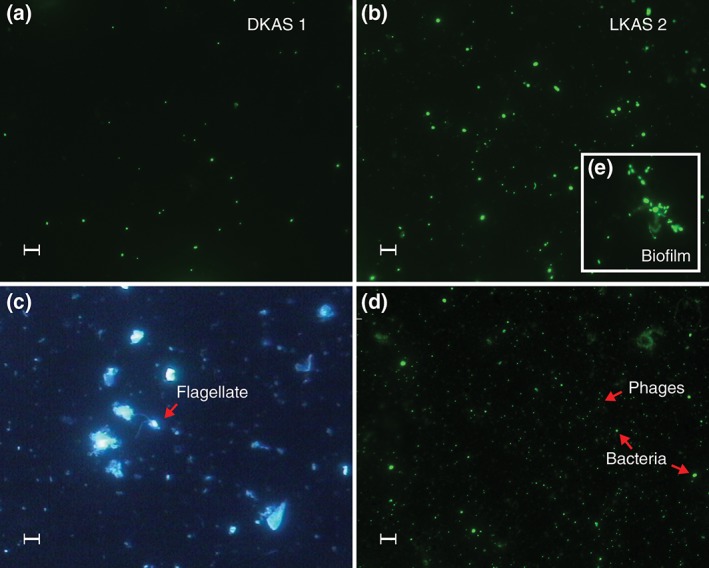
Epifluorescence microscope images of microbial communities in spring water of the limestone karst aquifer 2 (LKAS2) and the dolomite karst aquifer 1 (DKAS1). Panel (a) shows typical prokaryotic cells in the spring water of the DKAS1 system at 1000× magnification, stained with SYBR® Gold nucleic acid gel stain; panel (b) shows prokaryotic communities in the spring water of LKAS2 (large picture), and an example of a detached “biofilm‐floc” (inset, e); the red arrow in panel (c) highlights an example of an heterotrophic nanoflagellate in LKAS2, detected by DAPI fluorescence staining at a magnification of 400×; panel (d) shows an image of bacteriophages, also at 1000× magnification and stained with SYBR® Gold nucleic acid gel stain. The scale bars indicate a width of 5 μm (a, b, and d) and 12.5 μm (c), respectively. The analysis was made with a Nikon Eclipse 80i fluorescence microscope using fluorescence dyes for the detection of microbial cells or phages after they were filtered on filters

A first study investigated the occurrence and dynamics of total prokaryotic^1^ cell counts (Figure 3a,b) by EM in the NCA alpine spring water from two hydrogeologically contrasting but closely located karst aquifers over several years (Farnleitner et al., 2005; Wilhartitz et al., 2009). Spring DKAS1 represents a dolomite–limestone karst aquifer featuring a high average water residence time and a relatively constant flow (Q_min_/Q_max_ discharge ratio of 1:1.6), whereas spring LKAS2 drains a typical limestone karst aquifer with a very dynamic hydrological regime and discharge (Q_min_/Q_max_ discharge ratio of 1:40; see section 4 ‘selected model catchments’ for more details). The detected levels of total cell counts (TCC) and its variations in the spring water reflected the different hydrogeological situation of the systems. DKAS1 showed a relatively stable TCC that only ranged from 8 to 20 × 10^3^ cells per mL (*n* = 74) during the almost 5‐year study period (from year 2001 to 2005, Table 1). In 2005, EM counts were paralleled by FCM counts. TCC obtained by FCM and EM followed the same pattern throughout the whole year and averaged to 1.4 × 10^4^ cells per mL for both methods (Wilhartitz et al., 2013). The TCC did not reveal any relationship to the determined hydrological and physicochemical parameters (e.g., discharge, turbidity, electrical conductivity, and spectroscopic absorption at 254 nm). Principal component analysis indicated a biological component in the DKAS1 aquifer (e.g., comprising bacterial biomass and activity), which was largely independent from the prevailing discharge regime (Wilhartitz et al., 2009). In contrast, the TCC from the dynamic LKAS2 showed close association to the prevailing hydrological conditions and measured water quality characteristics (Farnleitner et al., 2005; Wilhartitz et al., 2009, 2013). The TCC ranged from 26 to 107 × 10^3^ cells per mL (*n* = 74) during the investigation period, with the lowest concentrations during the winter season and the peak values connected to high‐discharge events during summer season. In addition, a detailed investigation of a high‐discharge event at LKAS2 in summer 2008 revealed TCC up to 387 × 10^3^ cells per mL (data not published). Similarly to DKAS1, EM counts gave similar results as FCM counts at base flow conditions (discharge <4,500 L s^−1^) in 2005. However, during rainfall events absolute numbers diverged, with FCM counts being higher. As a result, averaged TCC in LKAS2 were 4.4 × 10^4^ cells per mL for EM and 7.8 × 10^4^ for FCM (Wilhartitz et al., 2013). In agreement with the TCC results, also prokaryotic cell volumes and sizes, cell biomasses and cell shapes (morphotypes) reflected the hydrogeological differences between the DKAS1 and the LKAS2 site (Farnleitner et al., 2005; Wilhartitz et al., 2009, 2013). Compared with LKAS2, DKAS1 revealed smaller cell volumes and increased proportions of coccoid cells (Figure 3a,b). It could be shown that bacterial cells represented the dominant fraction in the spring water of the LKAS2, LKAS4, LKAS8, and the DKAS1, whereas archaeal cells contributed only a small fraction (≤12%) of the total prokaryotic cell counts (Wilhartitz et al., 2007). A few years later, the observations from LKAS and DKAS1 were also supported by a study investigating the TCC in karst springs within the Swiss Jura Mountains and the Alpine Chain (National Groundwater Monitoring programme of Switzerland) based on FCM analysis (Sinreich et al., 2014). There, the TCC ranged from 3 to 500 × 10^3^ cells per mL of spring water, well reflecting the range of various other investigated hydrogeological systems (Table 1). From this result, Sinreich et al. (2014) concluded that the TCC represents a valuable intrinsic parameter for karst aquifer characterization. Moreover, they suggested a hydrogeological‐microbiological classification system of karst springs based on the observed TCC ranges (i.e., 10^3^–10^4^, 10^4^–10^5^, 10^5^–10^6^ cells per mL). This system is also supported by TCC measurements from other available investigations of alpine karst springs or cave pools that fall within the discussed range of reported cell numbers (Table 1). In this context, it has to be considered that TCC results in different studies may differ depending on the method used. As mentioned above, for DKAS1 and LKAS2, EM and FCM results were similar under baseflow conditions, but higher results were obtained with FCM during rainfall events. TCC results obtained by EM also depend on the used fluorochrome (Seo, Ahn, & Zo, 2010). It is known that, for example, 4′,6‐diamidino*‐*2*‐*phenylindole (DAPI) staining tends to underestimate TCC in comparison to acridine orange (Seo et al., 2010). In the discussed studies from the NCA region (Wilhartitz et al., 2009, 2013), acridine orange (Merck, Darmstadt, Germany) was used, while SYBR Green (Invitrogen, Waltham, MA, USA) was used for FCM. For an alluvial groundwater aquifer system, we compared EM SYBR Gold staining (instead of Acridin orange) with FCM SYBR Green and found FCM values being on average 26% lower than the values obtained by EM, with a highly significant correlation between both methods (*r* = 0.91, *n* = 138, *p* < 0.001; unpublished data). Besides, it has to be highlighted that the spring water of alpine karst aquifers also contains a substantial number of bacteriophages (i.e., viruses specifically infecting prokaryotic cells) as well as protozoan organisms, although the latter could be observed only in very low concentrations (Wilhartitz et al., 2013; cf. Figure 3c,d). Apart from the information given by Wilhartitz et al. (2013), there is currently hardly any information available on the intrinsic phage or protozoan community composition in alpine karst spring water.

**Table 1:** A summary table on total prokaryotic cell concentrations (TCC) in water from available studies on distinct karst aquifers in Europe

Study site	Geology	Altitude	Total cell counts (TCC) [10^3^ cells/mL]		Study period	Season	*n* value	Method (dye)	Geographic location (country)	Reference
			Mean/median^a^	Range						
Spring	Limestone karst (LKAS)	Alpine	63.0^a^	44–107	1 year	All seasons	*n* = 15	EM (AO)	Northern Calcareous Alps (Austria)	Farnleitner et al. (2005)
			44.4^a^	27–70	2 years	All seasons	*n* = 19–25	EM (AO)		Wilhartitz et al. (2009)
			50.6^a^	26–85	3 years	All seasons	*n* = 40	EM (AO)		Wilhartitz et al. (2013)
	Dolomite karst (DKAS)		14.8^a^	13–20	1 year	All seasons	*n* = 15	EM (AO)		Farnleitner et al. (2005)
			13.1^a^	11.2–19	2 years	All seasons	*n* = 19–25	EM (AO)		Wilhartitz et al. (2009)
			13.2^a^	8–19	3 years	All seasons	*n* = 40	EM (AO)		EM (AO)
	Limestone and dolomite karst springs		27.0	21–34	1 year	Winter to summer	*n* = 56	EM (DAPI)		Wilhartitz et al. (2007)
Spring	Karst hydrogeological settings	Subalpine and alpine		3–500	1 year	Autumn and spring	*n* = 14	FCM (SG I)	Jura Mountains and Alpine Chain (Switzerland)	Sinreich et al. (2014)
Spring	Limestone karst (highly karstified)	Low mountain range		19–650	1 year	Spring and autumn	NA (every 45 min)	FCM (SG I)	North western Switzerland (Switzerland)	Page et al. (2017)
				21–389						
				15–339						
Ground water	Limestone karst (German Muschelkalk)	Lowland karst (upper aquifer)		11–94	1 year	Spring to autumn	*n* = 8	EM (SG II)	Hainich national park (Germany)	Opitz et al. (2014)
		Lowland karst (lower aquifer)		2.7–380						
				12–370						
Pool	Limestone karst	Epiphreatic subsurface karst (950 m)		270–520	1 year	Autumn and winter	*n* = 8	EM (DAPI)	Bärenschacht cave in Bernese Oberland (Switzerland)	Shabarova and Pernthaler (2010)
				100–290			*n* = 6	FCM (SG I)		Shabarova, et al. (2013)

*Note*. FCM = flow cytometry; EM = epifluorescence microscopy (used fluorescence dye in the respective study); NA = information not available; DAPI = 4′,6‐diamidino*‐*2*‐*phenylindole; SG I = SYBR® Green I (Invitrogen); SG II = SYBR® Green II (Invitrogen); AO = acridine orange.

a Indicates statement of “median” instead of “mean” concentrations.

### 5.2 Microbial activity, suspended versus attached growth and biogeochemical significance

Other important questions relate to the potential impact of the microbiota on the “self‐purification” of spring water quality and on carbonate geochemistry (Gray & Engel, 2013). In this respect, prokaryotic activity measurements performed in NCA spring water from DKAS1 and LKAS2 based on sensitive analytical isotope approaches revealed extremely low heterotrophic production rates (i.e., 0.72 to 82 pg carbon L^−1^ hr^−1^ for DKAS1, and 6 to 900 pg carbon L^−1^ hr^−1^ for LKAS2) during two annual cycles of investigation (Wilhartitz et al., 2009); these measured production rates were among the lowest values ever measured for aquatic habitats (Eiler et al., 2003; Kirschner & Farnleitner, 2005; Laybourn‐Parry, Quayle, Henshaw, Ruddell, & Marchant, 2001). The combination of activity measurements, microscopy, and specific cell‐labeling techniques (i.e., catalyzed reporter deposition‐FISH‐microautoradiography) further demonstrated that, on average, only 7% (range 3–14%) of the observed TCC in the spring water were active, with very long generation times of up to 684 days (Wilhartitz et al., 2009). These extremely low prokaryotic activity rates in the suspended compartment of spring water from the base flow component of DKAS1 and LKAS2 also suggest a potentially high biostability^2^ for water supply purposes. However, it has to be emphasized that biostability is defined in an operational context and all possible changes during water abstraction, treatment, disinfection, and supply in the distribution net must be considered (Chowdhury, 2012; Liu, Verberk, & Van Dijk, 2013; Van Der Kooij, 2000; Zhang, Oh, & Liu, 2017). The biostability strongly depends on the type, characteristics, and management of the water supply system and thus has to be determined for the specific situation.

**Figure 4:**
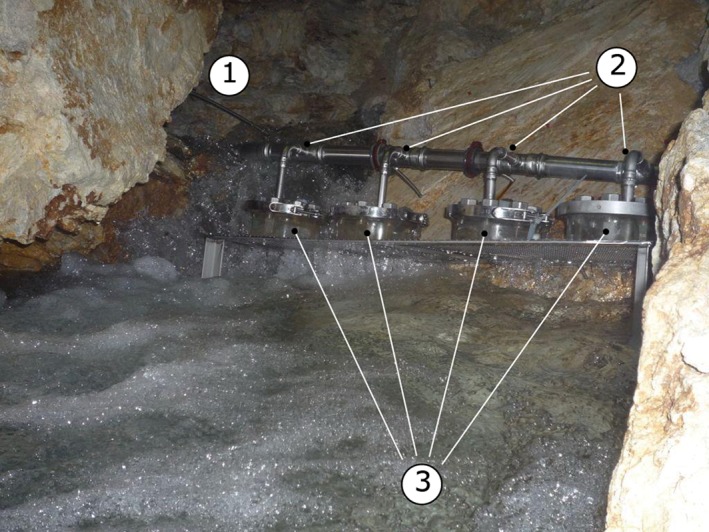
Hardware setup for the investigation of microbial colonization. Experiment examining rock surfaces under dark in situ incubation conditions; (1) The water inlet directly from spring DKAS1; (2) the inlets to each of the four inert incubation chambers containing the freshly sliced autochthonous rock disks; and (3) the actual incubation chambers. All parts were built of corrosion‐resistant and inert steel material

**Figure 5:**
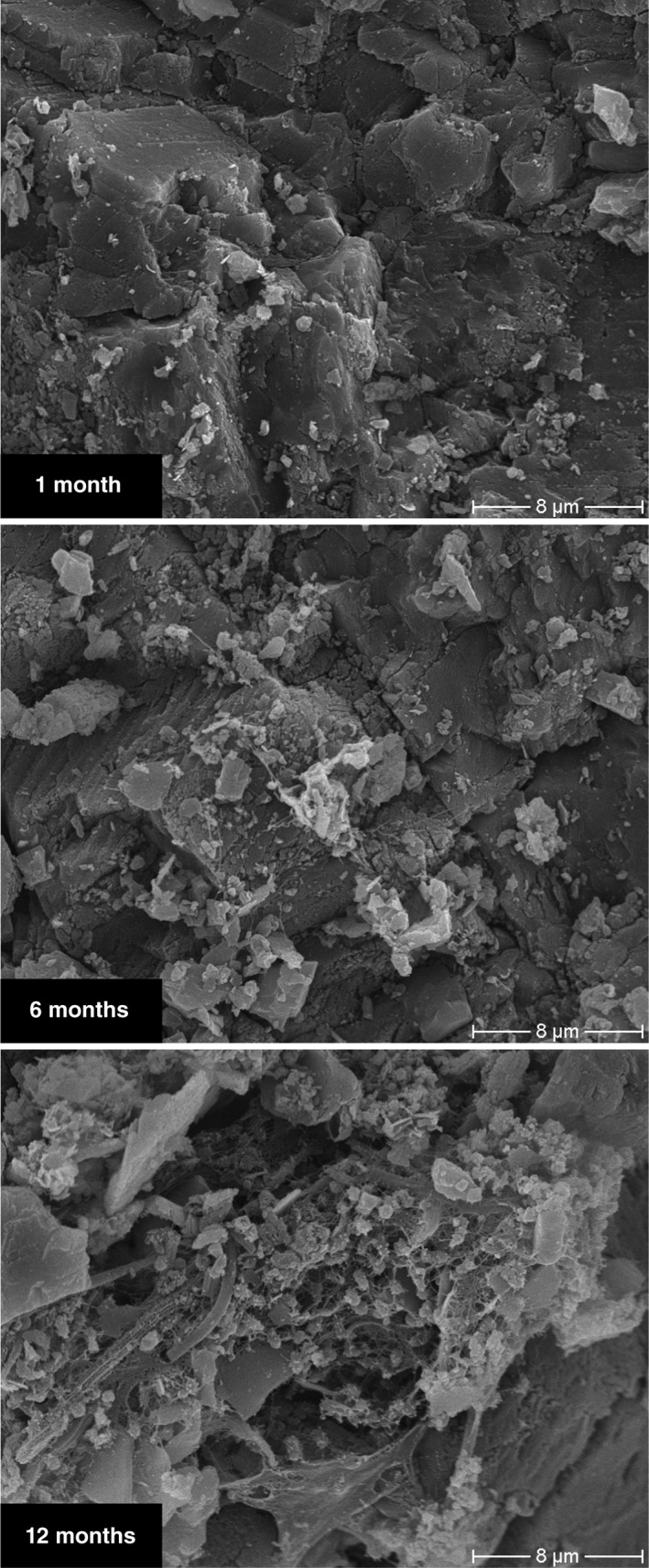
Previously unpublished scanning electron microscope (SEM) images from in situ colonization experiments in a spring housing under dark in situ conditions, using freshly sliced natural limestone disks prepared from rock material as found in the DKAS1 catchment (“Wettersteindolomit”) as growth substrates. The hardware setup for the incubation experiment is given in Figure 4. The pictures show the change of the rock surface during 1 year of incubation in the DKAS1 spring water (1, 6, and 12 months). The increasing amount of particle accumulation or formation on the rock surfaces can be seen. Additionally, the first indications of organic structures became visible (e.g., 12 months). The scale bars indicate the extent of magnification

In contrast to the extremely low growth rates of cells suspended in native spring water, the activity measurements of sediments recovered from DKAS1, LKAS2, LKAS4, and LKAS8 on average revealed a 1‐million‐fold (10^6^) higher heterotrophic production rate per volume when compared with measurements in the overlying spring water. These observations at the NCA springs are in agreement with those of previous studies of porous aquifers, which often reported that groundwater samples do not accurately reflect the aquifer microbiology due to a high ratio between the rock and sediment attached and the suspended cells in the water column (Alfreider, Krössbacher, & Psenner, 1997; Lehman, 2007). Hence, the activity rates determined by Wilhartitz et al. (2009) highlight the importance of rock or sediment‐surface‐associated microbial processes within karst aquifers (e.g., at sediment particles, fractures, and conduits) and suggest their role on self‐purification and geochemical processes. In situ colonization experiments with natural limestone and dolomite rocks from LKAS/DKAS placed directly in the spring (Figure 4) first indicated that microbial colonization is indeed supported within the sediment and rock structures of alpine karst aquifers (Figure 5). However, more investigations are required to substantiate these initial findings at NCA spring water and to better understand the processes associated with rock and sediment surfaces in alpine karst aquifers.

### 5.3 Elucidating the microbial community structure by genetic analysis

To further characterize the potential function of the observed bacterial communities in alpine spring water, basic information on their composition and structure is of interest. Therefore, PCR‐dependent fingerprinting based on a ribosomal gene (i.e., PCR‐DGGE profiling of the bacterial V3‐16S‐rRNA gene) was performed with NCA spring water from DKAS1 and LKAS2 during 2000 and 2001. The bacterial community profiles revealed remarkable stability for each spring water type, being easily classifiable as either DKAS1 or LKAS2 DGGE‐profile types (Farnleitner et al., 2005). Another study that applied a comparable methodology to spring water from a catchment in the Swiss Jura Mountains supported the high temporal stability of bacterial populations in the base flow component of alpine karst spring water (Pronk et al., 2006; Pronk, Goldscheider, & Zopfi, 2009). Because of its high average water residence time (~20 years) and extremely low vulnerability to microbial pollution from surface soil layers, the spring water of DKAS1 was also subjected to ribosomal gene sequencing. The retrieved nucleic acid sequences revealed low similarities when compared with sequences in available databases (Pruesse, Peplies, & Glöckner, 2012), indicating the existence of unique bacterial communities at the DKAS1 habitat (Farnleitner et al., 2005). Some of these sequences also indicated the existence of bacterial populations with chemolithoautotrophic activities. For example, the sequences sharing the highest similarity with the most abundant sequence were affiliated with the family *Nitrospiraceae*, which is a physiologically highly diverse family that includes nitrite or ferrous iron oxidizers (Daims, 2014). A few years later, *Nitrospiraceae*‐like sequences were also reported in several other studies on the bacterial community composition in pristine karstic or granitic alpine aquifers or cave systems (Herrmann et al., 2015; Konno et al., 2013; Kostanjšek, Pašić, Daims, & Sket, 2013; Pleše et al., 2016; Pronk et al., 2009; Wu, Xing, & Zhou, 2010).

### 5.4 Defining the AMEC versus TMEC concept

**Figure 6:**
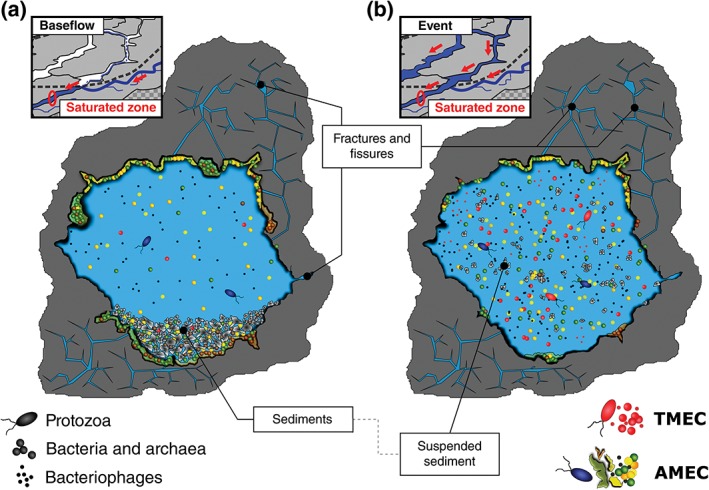
Schematic illustration of a cross‐sectional view into a conduit in the phreatic zone of a limestone karst aquifer (cf. Figure 1) during base‐flow conditions (a) and under increased discharge conditions due to rapid surface water input during a precipitation event in the catchment (b). The figures indicate the postulated distribution of the most important microbial compartments in these systems, including the suspended fraction in the water column as well as the fraction attached to the conduit walls or alluvial sediments. The proposed differences between the base‐flow and the high‐discharge event situation are (1) an increased mobilization of rock biofilm‐ and sediment‐associated microbes due to the increased shear stress and (2) increased numbers of surface‐associated and transiently occurring microbes (red) infiltrating from the surface (for details, see main text). Red‐colored microbes (bacteria, archaea, protozoa, bacteriophages) represent the transient microbial endokarst communities (TMEC); all other colors (yellow, orange, green, blue) show the autochthonous microbial endokarst community (AMEC), whereas the diversity in colors shall reflect the proposed diversity of these naturally occurring microbes

By bringing together all the results recovered from the NCA LKAS/DKAS sites, the so‐called autochthonous microbial endokarst community (AMEC) versus transient microbial endokarst community (TMEC) concept was formulated for alpine spring water (Farnleitner et al., 2005). The aim of this concept is to predict the proportion of AMEC versus TMEC and its typical range of variation in spring water according to the catchment and the hydrogeology of the investigated system. To facilitate its correct understanding and interpretation (Brannen‐Donnelly & Engel, 2015; Wilhartitz & Farnleitner, 2010), the concept is described in more detail in the following section. Microbes that are unable to proliferate and unable to form stable populations within the karst aquifer are considered TMEC. Their occurrence in spring water is considered to be controlled by two factors: (a) the extent of microbial cell input from external environments (e.g., direct input by swallow holes, surface runoff during precipitation‐triggered events, seepage from the soil–plant–vegetation compartment) and (b) by the governing physical and biological factors of microbial cell transport through the aquifer toward the spring (e.g., convection, dispersion, dilution, attachment, straining, persistence, and grazing). In contrast, the AMEC is considered to be a collection of microbes that possess the physiological capabilities to build sustainable populations by growth and reproduction under the prevalent conditions in the karst aquifer. AMEC cells may grow in the water phase (suspended mode) or attached to rock or sediment‐surfaces (biofilm mode; Figure 6a). During situations of increased discharge, sediment and rock‐attached cells are thought to be increasingly detached and mobilized and show up in the suspended fraction (Figure 6b). At this point, we want to emphasize that the definition of the AMEC is not linked to an endemic evolutionary origin in karst systems. Instead, these microbes are likely also occurring in other habitats such as alpine soils, streams, or lakes. The question whether endemic AMECs do exist in karst aquifers (in a sense that they evolved and occur only within this habitat) is still speculative and subject to further research (Griebler & Lueders, 2009).

Investigations applying cutting edge RNA/DNA HTS techniques to microbial communities (cf. Figure 2c) are needed to further evaluate and develop the AMEC/TMEC concept. Although not directly comparable, recent information from HTS investigations of stagnant cave pools in the epiphreatic zone of a karst system (Shabarova et al., 2013, 2014; Shabarova & Pernthaler, 2010) and an epigenic cave stream (Brannen‐Donnelly & Engel, 2015) indeed indicated the presence of stable and transient bacterial populations. For example, Shabarova et al. (2014) defined a bacterial “core” assemblage as the subset of those bacterial genotypes that were present in all samples during their temporal study of the bacterial community development in stagnant rock pools. On the contrary, these authors presumed that nonpersistent genotypes might have been introduced from interconnected environments and from other (e.g., anoxic) endokarst habitats, which supports the idea of a TMEC (Shabarova et al., 2014).

## 6 NEW STRATEGIES TO MONITOR AND MANAGE MICROBIAL FECAL POLLUTION

During precipitation events and therewith associated high‐discharge situations, microbes from soil, sediment, plant, animal and human habitats (cf. Figure 1) may be flushed into alpine karst aquifers via the overlaying soil surface layers. Depending on the input load, fate, and mobility (see also AMEC/TMEC hypothesis definition above), these cells may finally also be detected in the spring water, as also reflected in an increase of copiotrophic microbes^3^ measurable by HPC techniques (Figure 6; Farnleitner et al., 2005). If this water is used for water supply, nutrients from catchment surface‐associated habitats can also decrease the biostability of the spring water, potentially resulting in undesirable changes of water quality related to taste, odor, or turbidity of the water (Chowdhury, 2012; Van der Kooij et al., 2013). As intestinal pathogens can occur in extremely high concentrations in human and animal excreta, microbial fecal pollution represents the most critical water quality hazard in alpine spring water (Hrudey & Hrudey, 2004; Kralik, 2001). Depending on the hydrogeology and the hydrological situation of the considered aquifer and spring systems, fecal pollution can occur extremely quickly, and the corresponding pollution levels can increase by more than several orders of magnitude during an precipitation‐triggered high‐discharge event situation (Sinreich et al., 2014; Stadler et al., 2008). Despite its high relevance for water supply and public health, until recently, microbial fecal pollution in spring water was considered a “black box” phenomenon. Consequently, this lack of knowledge also hindered target‐oriented water quality management.

### 6.1 The framework for integrated fecal pollution analysis and management

**Figure 7:**
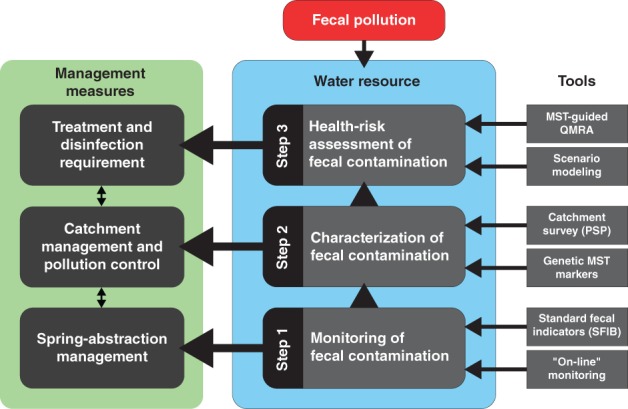
Schematic illustration of the suggested “framework for integrated fecal pollution analysis and management” to guide spring water quality management in accordance with the World Health Organization criteria (for details, see main text). Target‐oriented catchment protection and optimized spring water‐abstraction management helps to minimize the required treatment and disinfection efforts and to maximize the biostability of the spring water to provide supply with high‐quality drinking water. The three interacting levels (“three‐step approach”) include (1) the monitoring of potential fecal pollution, (2) the characterization and identification of potential fecal pollution sources in the catchment, and (3) the assessment of the actual health risk from human exposure (drinking), in relation to the fecal source(s). MST, microbial source tracking; QMRA, quantitative microbial‐risk assessment; PSP, pollution source profiling; SFIB, standard fecal indicator bacteria

To promote advanced spring water quality management for the 21st century, a new strategy was recently proposed based on the NCA LKAS and DKAS research; this strategy is the so‐called “framework for integrated fecal pollution analysis and management” (Farnleitner et al., 2018; Stalder et al., 2011a). Three interacting levels (“three‐step approach”) characterize the backbone of the concept (Figure 7), with relevance to the following issues: (a) is there a problem with fecal pollution? (b) if yes, what is the reason for it? and (c) what is the actual health risk related to the fecal source(s) that contribute to the observed pollution? The suggested framework can also be referred to as a “bottom‐up approach” because it starts at the most general level (i.e., general pollution monitoring, including all types of fecal pollution sources from humans, life stock, and wildlife) and becomes more specific as it proceeds from the bottom to the top of the diagram (i.e., looking for the responsible pollution source(s) and the associated health risks for the consumer of the drinking water). The three steps will be explained in detail below.

*Step 1: Detection and online monitoring of microbial fecal pollution*

Microbiological water quality monitoring has been based on the detection of standard fecal indicator bacteria (SFIB) for more than 100 years. SFIB can be easily detected by cultivation‐based standard procedures (e.g., ISO 9308‐1 for *Escherichia coli*, *E. coli*, and ISO 7899‐2 for intestinal enterococci; International Organisation for Standardisation, 2000a, 2000b). The most basic requirements of sensitive fecal indicators are the occurrence of these bacteria in high concentrations in the excreta of humans and warm‐blooded animals and their inability to replicate outside of the intestinal environment. However, especially regarding the latter, the capacity of SFIB to indicate fecal pollution in water resources has been increasingly questioned during the past decade (Ishii & Sadowsky, 2008). The reason for this debate is the suggested existence of “naturalized populations” of commonly used SFIB, which are thought to persist and proliferate outside the intestinal environments, including in sediments and soils (Brennan, Abram, Chinalia, Richards, & O'Flaherty, 2010; Byappanahalli, Nevers, Korajkic, Staley, & Harwood, 2012; Derry & Attwater, 2014; Ishii & Sadowsky, 2008). Thus, the SFIB were rigorously evaluated for the LKAS/DKAS systems, as there was hardly any available information on their performance characteristics in alpine catchments. These investigations clearly demonstrated that SFIB can indeed be reliably used for sensitive fecal pollution monitoring at alpine karst water resources (Reischer et al., 2008; Stadler et al., 2010). Moreover, for the LKAS2 system, the microbial fecal pollution dynamics could also be elucidated in a high‐resolution mode using automated high‐discharge event‐triggered sampling for SFIB and other microbial indicators, combined with online monitoring of discharge and physicochemical parameters (Stadler et al., 2008).

**Figure 8:**
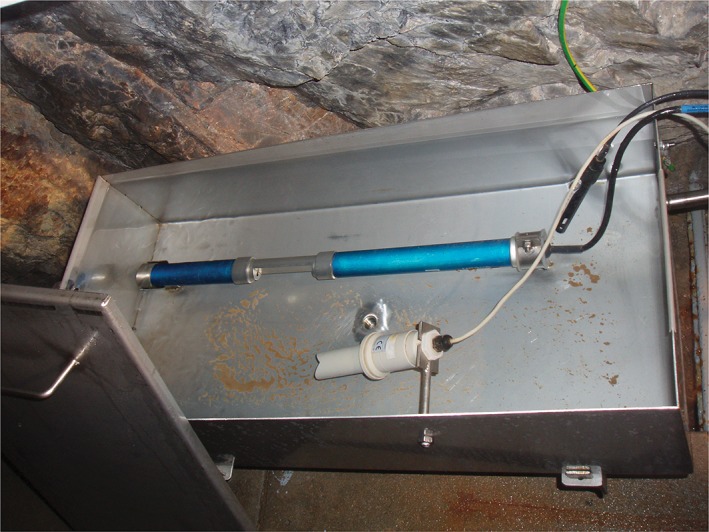
Installation of an optical s::can probe (blue device; scan Messtechnik GmbH, Wien, Austria) at a spring environment for near‐real‐time online measurement of turbidity and SAC254 (spectral absorption coefficient at 254 nm)

Rapid online monitoring of fecal pollution events in spring water would be highly desirable as an extension to traditional SFIB detection, as cultivation usually takes more than one working day. For this reason, automated enzymatic detection, using β‐D‐glucuronidase (GLUC) activity, was evaluated as a tool for near‐real‐time online fecal pollution monitoring. GLUC activity was previously suggested as a rapid biochemical indicator of fecal pollution in rivers and estuaries (Farnleitner et al., 2001; Farnleitner, Hocke, Beiwl, Kavka, & Mach, 2002; Tryland & Fiksdal, 1998) but has not been applied in groundwater habitats. A two‐year investigation at the LKAS2 site demonstrated that robust automated online determination of enzymatic microbial activity rates directly at alpine spring water locations can be successfully implemented based on the currently available technology (Ryzinska‐Paier et al., 2014; Stadler et al., 2016). However, contrary to expectations, GLUC activity did not qualify as a rapid surrogate parameter for cultivation‐based SFIB pollution. Thus, further investigations are needed to clarify the actual indication capacity of GLUC (and other enzymatic alternatives) as a rapid means for fecal pollution monitoring in such habitats (Ender, Goeppert, Grimmeisen, & Goldscheider, 2017; Ryzinska‐Paier et al., 2014). As an alternative to complex biochemical online detection, physicochemical online measurements may also support the real‐time detection of surface‐associated fecal pollution influence in spring water. Indeed, the spectral absorption coefficient at 254 nm (SAC254) could be identified as a real‐time early‐warning proxy for fecal pollution in the LKAS systems (Figure 8). This finding was based on a statistical time‐series analysis using an autosampling setup that generated “high‐resolution” data series for the LKAS2, LKAS4, and LKAS6 sites during high‐discharge events occurring in summers of 2005–2008 (Stadler et al., 2008). Interestingly, the SAC254 also increased between 3 and 6 hr earlier (so called “lead‐time”) than the potential onset of fecal pollution, irrespective of the studied high‐discharge event (Stadler et al., 2010). The physical online monitoring parameter turbidity, as a rapid proxy for surface‐associated fecal pollution, was demonstrated on a spring in the Swiss Jura Mountains. Pronk et al. (2006) and Pronk, Goldscheider, and Zopfi (2007) applied the continuous detection of particle‐size distributions and showed that a relative increase of finer particles (0.9–10 μm) were associated with surface influence and the potential contamination with fecal indicator bacteria. It is important to note that the ability to predict the occurrence of fecal pollution by a proxy parameter (or a combination of several parameters) is closely linked to the characteristics of the habitat. A proxy parameter must not be transferred to other situations without rigorous evaluation, whether such an application is justified.

*Step 2: Characterization and identification of fecal pollution sources*

The detection of SFIB indicates and quantifies fecal pollution in spring water. However, SFIB enumeration does not hold any information about the sources responsible for fecal pollution (i.e., human, life stock, or wildlife). Thus, a combination of pollution source profiles (PSP; i.e., generating information on potential pollution sources within a specific catchment) and host‐associated genetic fecal markers (i.e., specific DNA fragments of host‐associated gut microbes) were developed and evaluated for MST (i.e., generating information on the origin of fecal pollution directly from the spring water) in the LKAS catchments. The quantitative assessment of potential fecal pollution sources in the respective catchment—also referred to as PSP (Figure 7)—facilitated the formation of hypotheses regarding the suspected pollution sources of spring water contamination (Farnleitner et al., 2011; Reischer et al., 2011, 2013). For example, as much as 99.9% of intestinal *E. coli* populations daily produced and deposited in the LKAS6 environment could be allocated to wildlife (42.4%) or livestock ruminant (57.6%) fecal excreta emissions. In contrast, the availability of human fecal pollution sources was estimated to be negligible within the same catchment (Farnleitner et al., 2011).

To test the fecal pollution source hypothesis suggested by the PSP, host‐associated genetic fecal markers were developed, evaluated, and subsequently applied directly to the spring (Farnleitner et al., 2011). Based on intestinal *Bacteroidetes* populations^4^, qPCR approaches could be successfully developed for the sensitive detection of a ruminant‐associated genetic fecal marker (i.e., the BacR approach [Reischer, Kasper, Steinborn, Mach, & Farnleitner, 2006]) and a human‐associated genetic fecal marker (i.e., the BacH approach [Reischer, Kasper, Steinborn, Farnleitner, & Mach, 2007]). For example, the BacR qPCR approach detects, on average, 4.1 × 10^9^ marker molecules per gram of ruminant feces, permitting the ultrasensitive detection of approximately 2 ng of ruminant feces per analyzed water sample (Reischer et al., 2006). Using hydrology‐guided sampling and multiparametric analysis (so called “nested sampling” design), the developed BacR/BacH marker allowed the determination of the relevant fecal pollution sources of spring water contamination (Farnleitner et al., 2011; Reischer et al., 2008, 2011). For example, the hypothesis that ruminant animals were the dominant sources of fecal pollution in the LKAS6 catchment was clearly confirmed. In addition, *E. coli* contamination could be predicted based on the measured concentrations of the BacR marker at the considered spring (Farnleitner et al., 2011).

In general, the quantification of host‐associated fecal markers in water samples by PCR methodologies is a very recent development, and many further applications are likely to follow in the near future. At the time the NCA LKAS/DKAS project started, just a few PCR assays were available for the qualitative detection of genetic fecal markers (Bernhard & Field, 2000a, 2000b), and qPCR approaches had yet to be developed (Reischer et al., 2006, 2007; Stricker, Wilhartitz, Farnleitner, & Mach, 2008). Meanwhile, a vast number of new qPCR MST marker approaches has been developed during the last decade for various fecal pollution sources throughout the world (Wuertz, Wang, Reischer, & Farnleitner, 2011). Of these approaches, many are currently being evaluated for their application predominantly at surface water resources (Boehm et al., 2013; Hagedorn, Blanch, & Harwood, 2011; Mayer et al., 2016; Reischer et al., 2013). For (karst) ground water systems, however, just a few studies have been realized since the pioneering LKAS/DKAS work (Bucci, Petrella, Celico, & Naclerio, 2017; Diston, Robbi, Baumgartner, & Felleisen, 2017; Diston, Sinreich, Zimmermann, Baumgartner, & Felleisen, 2015; Ohad et al., 2015; Zhang, Kelly, Panno, & Liu, 2014). However, these studies already indicate the high potential of identifying host‐associated genetic qPCR markers to support traditional fecal pollution monitoring and to guide proactive and target‐oriented management in such systems.

*Step 3: Health‐risk assessment of microbial fecal pollution*

In most cases, water supplies require adequate primary disinfection to produce safe drinking water from karst aquifers (World Health Organization, 2017). The quantitative microbial‐risk assessment (QMRA) approach can help to determine the health risks associated with fecal pollution (Haas, Rose, & Gerba, 1999, 2014). The QMRA approach also supports the estimation of the required extent of pathogen reduction during disinfection to ensure the required safety level for water consumption (Schijven, Derx, de Roda Husman, Blaschke, & Farnleitner, 2015; Schijven, Teunis, Rutjes, Bouwknegt, & de Roda Husman, 2011). The QMRA relies on the appropriate choice and quantification of reference pathogens (RP; Haas et al., 1999). Commonly used RP include representative pathogens from enteric bacteria (e.g., *Campylobacter* sp.), enteric parasites (e.g., *Giardia* sp.), and human enteric viruses (e.g., enteroviruses). As recently demonstrated, many more zoonotic agents, potentially occurring in mountainous alpine catchment areas, can be relevant to human health (Stalder et al., 2011a, 2011b).

The suggested “bottom‐up” approach (Figure 7) uses information from Step 2 to guide the selection of appropriate RP for Step 3 by integrating results from the PSP and MST. For example, this approach enabled researchers to focus on zoonotic pathogens in the LKAS2 system, specifically evaluating the quantitative occurrence of *Cryptosporidium* sp. and *Giardia* sp. directly in the fecal pollution sources at the catchment and in the spring water during high‐discharge events (Farnleitner et al., 2018).

## 7 CONCLUSION—STATUS QUO AND FUTURE RESEARCH NEEDS

The presented studies from the NCA area and from studies performed by other groups abroad Austria shed considerable light on the microbiology in the spring water from alpine karst aquifers. It could be demonstrated that the hydrogeology and catchment situation of a given system has a strong impact on the microbiological characteristics of the spring. According to their hydrogeological settings, alpine karst springs behave as “individuals” and thus require specific adaptation of their management when used for water supply.

Investigations demonstrated that even spring water with a high water residence time and absence of immediate surface influence is far from being sterile (cf. DKAS2) and harbors characteristic natural (intrinsic) microbial communities. Observed TCC, sizes, volumes and morphotypes are strongly impacted by the hydrogeological situation of the aquifer. In this regard, spring water has to be defined in terms of basic physical (e.g., temperature), chemical (e.g., inorganic ion composition), and biological (e.g., numbers of TCC) water quality characteristics in future concepts. To highlight the ecosystem character of alpine karst springs, the AMEC/TMEC hypothesis has been suggested for the investigated NCA DKAS and LKAS systems. It should be noted that further research activities are needed to evaluate this concept at various locations and in different situations. To date, only very little molecular biological information is available. However, the application of HTS (cf. Figure 2) is likely to provide an adequate tool to elucidate this question and to foster further research activities on the microbiome of spring water of alpine karst aquifers. In the long run, HTS and bioinformatics may use the total amount of biological information stored in the analyzed water samples for a more holistic water quality monitoring approach, with potential relevance for ecology (e.g., analyzing the ecological status), hydrology (e.g., using microbial spring communities as natural tracers), and public health and water supply (i.e., monitoring biohazard and biostability issues).

### Managing the surface‐related microbial pollution component

Depending on the aquifer system, a unique selection of measures such as catchment protection, water‐abstraction management (using the water for drinking water production only when it shows appropriate raw water quality) and sufficient treatment (including disinfection) must be combined to guarantee a high‐quality drinking water supply. In this context, the suggested framework for microbial (fecal) pollution analysis and management (Figure 7) provides a conceptual basis to select optimal management solutions for specific spring systems and water supply situations that are in accordance with the health‐based water quality safety criteria of the World Health Organization (World Health Organization, 2017), as to ensure a maximum tolerable infection risk per person and time during drinking water consumption (Farnleitner et al., 2018). The framework also highlights that target‐oriented catchment protection, and optimized spring water‐abstraction management helps to minimize the required treatment and disinfection efforts to keep the final drinking water close to natural conditions for a safe and high‐quality drinking water supply. Spring‐abstraction management also helps to reduce the potential input of nutrients and copiotrophic microbes, optimizing the biostability of the water during treatment and distribution. The philosophy of the suggested framework is in accordance with the guidelines of the Austrian Codex Alimentarius, which states that the greatest effort should be put into the protection and abstraction of raw water in order to keep the required treatment and disinfection as low as possible (Federal Ministry of Health and Women's Affairs, 2017).

Online water quality monitoring, MST based on genetic fecal marker enumeration, and MST‐guided QMRA are suggested key analytical tools to realize the proposed framework for the spring environment. Near‐real‐time detection of fecal pollution needs to be fast and sensitive and able to follow rapidly changing water quality characteristics. However, as demonstrated for the GLUC approach, such techniques still demand further research effort and development. Whether the recently emerging online detection systems for TCC enumeration (Besmer et al., 2014, 2016; Hammes et al., 2008; Hammes, Berger, Köster, & Egli, 2010; Van Nevel et al., 2017) also add significant value to this approach has yet to be evaluated. Irrespective of the applicability of currently emerging technologies, the general importance of microbiological online monitoring and automation (e.g., precipitation event‐triggered automated sampling) is likely to increase in the near future (Besmer et al., 2016; Højris et al., 2016; Ryzinska‐Paier et al., 2014; Stadler et al., 2016). The application of genetic MST markers also holds great promise for future water quality testing. Nevertheless, further research is indispensable. For example, the release of microbes from excreta and the mobility and persistence of fecal markers in the catchment and the aquifer are largely unknown. Furthermore, not all potential fecal sources in alpine areas are covered yet (e.g., alpine soil fauna). Moreover, to robustly guide reference pathogen selection for the QMRA (Farnleitner et al., 2018), the required sensitivity and specificity levels of the genetic fecal markers for specific source groups have to be evaluated in more detail. The QMRA is the least developed discipline within the presented framework. For example, there is a need for effective approaches to enrich microbial indicators and pathogens from large water volumes and to develop direct detection methods which differentiate between dead, viable or infectious pathogens. Last, but not least, modeling approaches linking hydrology, microbiology and infection and disease risks, as previously mainly developed for other systems (Epting, Page, Auckenthaler, & Huggenberger, 2018; Schijven et al., 2015), are increasingly needed. Such simulation tools will not only describe the status quo, but will also allow the evaluation of scenarios to design quality management strategies for a sustainable water supply from alpine karst aquifer springs of the future (Derx et al., 2016). An overview on open research questions and future development goals to fully realize the suggested framework for microbial pollution analysis and management is given in Table 2. As mentioned before, such a framework is not restricted to the fecal pollution component only, but considers the overall microbial quality of the spring water also including biostability aspects.

**Table 2:** Overview on open research questions and future development goals to fully realize the suggested framework for microbial pollution analysis and management

**Twenty three important research questions and development goals for spring water analysis and management**
*Sampling* What is the best technical approach to support microbiological autosampling to realize hydrological‐guided monitoring? What is the best approach to realize a combined sampling strategy for fecal pollution and biostability^2^ monitoring? What is the best approach for the enrichment of indicators and pathogens from large sampling volumes?
*Fecal pollution monitoring* What are the best complementing bacterial indicators to *Escherichia coli* (*E. coli*) and enterococci, supporting cultivation‐independent direct detection of total fecal pollution? What is the best suitable bacteriophage to indicate viral fecal pollution? What is the best combination of fecal indicators to indicate possible pollution with enteropathogenic bacteria, viruses, or protozoa?
*Microbial fecal source tracking (MST)* What is the persistence of the currently applied genetic fecal markers in alpine spring water? Do differences in the persistence between MST markers and standard fecal indicators occur? What is the degree of mobility of the currently applied genetic fecal markers in the catchment and the karst aquifer? How can the available genetic MST marker assays be extended and refined to differentiate all important fecal pollution sources (e.g., cattle versus sheep versus chamois versus deer)?
*Spring water‐abstraction management* What is the most suitable candidate parameter to facilitate sensitive and robust online monitoring of total fecal pollution? What is the most suitable candidate parameter to facilitate human‐associated (sewage) fecal pollution monitoring? What is the best approach to detect and differentiate recent fecal pollution versus aged fecal pollution at the spring water? What is the best approach to combine fecal pollution and biostability^2^ monitoring?
*Quantitative microbial‐risk assessment (QMRA)* What are the most suitable reference pathogens for QMRA? What is the significance of wild life versus livestock in terms of water contamination and health risk? What is the importance of zoonotic pathogens in terms of water contamination and health risk? What is the significance of soil biota in terms of water contamination and health risk? What is the most suitable modeling approach to link the catchment situation, spring water quality and QMRA? What are the required reduction levels for pathogens in spring water taking into account catchment management, hydrological characteristics, and spring water abstraction, to comply with World Health Organization water quality standards? What is the best approach to evaluate contamination‐ and health‐risk scenarios to support sustainable planning of water safety management infrastructure?
*Disinfection* What is the effect of Ultraviolet (UV) irradiation on the natural spring water bacterial community? What is the effect of UV irradiation on the nutrient availability and biostability^2^ in spring water?

## CONFLICT OF INTEREST

The authors declare no conflict of interest.

## RELATED WIREs ARTICLES

https://doi.org/10.1002/wat2.1120

https://doi.org/10.1002/wat2.1006

https://doi.org/10.1002/wat2.1146

## NOTES

1
Prokaryotic cells include bacterial and archaeal cell types. Besides eukaryotes (e.g. plants, animals) and bacteria, archaea represent the third kingdom of life on earth. Archaea show very similar cell shapes as compared to bacterial cells and thus cannot be distinguished by the TCC parameter.

2
The concept of biostability describes the ability to distribute and store water in a distribution system without detrimental quality changes by microbial growth based on the available substrates.

3
Copiotrophic microbes occur and grow in environments which are rich in nutrients and organic carbon. In contrast, oligotrophic microbes, such as postulated for AMEC, survive and grow at much lower nutrient and carbon concentrations.

4
*Bacteroidetes* are dominating members of the intestinal bacterial flora in humans and many animals. Some of the populations show also a strong host‐association (i.e. they predominately occur in a certain type of a host).
